# Acupuncture–antidepressant combinations for clinically diagnosed depressive disorders in China: a systematic review and network meta-analysis

**DOI:** 10.3389/fpsyt.2026.1805745

**Published:** 2026-10-20

**Authors:** Qiyue Qiu, Zheng Zhang, Weiwei Yang, Wan Yin Tew, Chenxi Liu, Mohammad Farris Iman Leong Bin Abdullah, Mun Fei Yam, Shichang Yang

**Affiliations:** 1 The Second Affiliated Hospital of Xinxiang Medical University, Xinxiang, Henan, China; 2 Department of Pharmacology, School of Pharmaceutical Sciences, Universiti Sains Malaysia, Gelugor, Penang, Malaysia; 3 The First Affiliated Hospital of Xinxiang Medical University, Xinxiang, Henan, China; 4 The Fifth People’s Hospital of Lingbao City, Lingbao, Henan, China; 5 Department of Psychiatry and Mental Health, Faculty of Medicine, Universiti Sultan Zainal Abidin, Kuala Terengganu, Terengganu, Malaysia

**Keywords:** acupuncture, adjunctive therapy, adverse effects, depressive disorders, network meta-analysis

## Abstract

**Introduction:**

To evaluate the short-term efficacy and safety of acupuncture–antidepressant combinations for clinically diagnosed depressive disorders among participants recruited in China using a network meta-analysis.

**Methods:**

PubMed, Embase, the Cochrane Library, Web of Science, CNKI, and Wanfang were searched from inception to February 2025 for randomised controlled trials of acupuncture–antidepressant combinations and relevant pharmacological or acupuncture-related comparators. The principal efficacy outcome was change in Hamilton Depression Rating Scale (HAMD/HDRS) scores; safety outcomes included nausea/vomiting, dry mouth, headache, and treatment discontinuation. Risk of bias was assessed using RoB 2. Random-effects Bayesian network meta-analyses were conducted in R version 3.6.0 using gemtc version 0.8.1. Mean differences or odds ratios are presented with 95% credible intervals (CrIs), together with posterior rank-position probabilities. A standardised mean difference (SMD)-based sensitivity analysis assessed the influence of pooling HAMD-17 and HAMD-24.

**Results:**

Seventy-seven trials (7,031 participants) were included in the systematic review. Within the principal connected eight-node efficacy network, manual acupuncture plus selective serotonin reuptake inhibitors produced the largest reduction versus SSRI monotherapy (mean difference −3.37; 95% CrI −3.86 to −2.89) and the highest probability of occupying Rank 8 (the most effective position). Electroacupuncture plus SSRIs was also associated with a greater reduction in HAMD scores than SSRIs alone (MD −2.98; 95% CrI −3.66 to −2.31). Manual acupuncture plus SNRIs also favoured combination treatment (MD −3.16; 95% CrI −6.03 to −0.28), although the interval was comparatively wide. The SMD sensitivity analysis retained benefit for both EA + SSRIs and MA + SSRIs relative to SSRIs, although their relative ordering differed from the raw-MD analysis. The available adverse-event networks did not identify statistically clear differences from SSRI monotherapy; however, sparse events and very wide CrIs limit the strength of this finding.

**Conclusion:**

Manual acupuncture plus an SSRI was associated with the largest short-term HAMD reduction in the primary raw-MD network. Relative treatment ordering differed in the SMD sensitivity analysis. The estimates should be interpreted cautiously because clinical heterogeneity, risk-of-bias concerns, sparse safety data, and limited follow-up preclude claims of equivalent safety or a preferred first-line strategy.

**Systematic Review Registration:**

https://www.crd.york.ac.uk/PROSPERO/View/CRD420251029584, identifier CRD420251029584.

## 1 Introduction

Major depressive disorder (MDD) is a common mental health condition, defined by a lasting low mood, loss of interest or pleasure, cognitive impairments, and disruptions in sleep or appetite. Severe cases can result in suicidal ideation or self-harming behaviour, posing a major burden on global public health (1). Bayesian age-period-cohort (BAPC) projections indicate that global age-standardized incidence (ASIR) and death rates (ASDR) will likely remain stable from 2020 to 2030, but case numbers may rise to 2,519.88 per 100,000 men and 3,835.11 per 100,000 women by 2030 (2). In China, over 50 million people—about 3.6% of the population—are estimated to suffer from depressive disorders (3).

First-line pharmacological treatments for MDD include several classes of antidepressants—SSRIs, SNRIs, NaSSAs, and TCAs—which primarily modulate central monoaminergic transmission. However, many patients do not respond adequately or discontinue treatment due to side effects including nausea, fatigue, variations in weight, or sexual dysfunction. These challenges have spurred interest in adjunctive or alternative therapeutic strategies.

Acupuncture, an essential aspect of traditional Chinese medicine, has attracted interest for its potential to alleviate depressive symptoms and improve emotional regulation. Its integration with pharmacological treatment is increasingly adopted in clinical practice and research. Emerging evidence indicates that acupuncture may exert antidepressant effects through modulation of neurotransmission, reduction of inflammation, and regulation of neuroimmune pathways. These findings support its role as a promising complementary intervention for MDD.

Previous meta-analyses of acupuncture for depression were limited by narrow eligibility criteria and small samples. Evidence specific to clinical practice in China also remains limited. We therefore conducted a network meta-analysis of randomised controlled trials undertaken in China that assessed acupuncture–antidepressant combinations and relevant comparators among participants with MDD or another clinically diagnosed depressive disorder in a defined clinical context.

This NMA integrates direct and indirect evidence within connected outcome-specific networks and describes the relative ranking of the evaluated interventions. Because the evidence base includes clinically heterogeneous depressive disorders and short follow-up periods, the estimates are intended to inform further research and cautious adjunctive clinical consideration rather than establish a treatment hierarchy for guideline adoption.

## 2 Methods

This systematic review and NMA adhered to the guidelines outlined by the Preferred Reporting Items for Systematic Reviews and Meta-Analyses (4) and its specific extension for Network Meta-Analyses of Health Care Interventions (PRISMA-NMA) (5). The study protocol was prospectively registered with the International Prospective Register of Systematic Reviews (PROSPERO) under the identifier CRD420251029584.

### 2.1 Search strategy

A comprehensive search was conducted in six electronic databases from inception to February 2025: PubMed, Embase, the Cochrane Library, Web of Science, China National Knowledge Infrastructure (CNKI), and Wanfang Data (**Supplementary Appendices S1**–**S6**). Searches were restricted to studies published in English or Chinese.

The strategy for searching utilized a mix of Medical Subject Headings (MeSH) alongside free-text keywords, which included but were not restricted to: “acupuncture,” “electroacupuncture,” “auricular acupuncture,” “moxibustion,” “acupoints,” “depression,” “depressive disorder,” “randomised controlled trial,” “antidepressants,” “SSRIs,” “SNRIs,” “NaSSA,” and “TCAs.” Database-specific adaptations were applied to optimize sensitivity and specificity. Detailed search strategies for all databases can be found in the **Supplementary Material**.

To reduce publication bias, we carefully reviewed the reference lists from all qualifying studies and pertinent systematic reviews. Moreover, we searched grey literature, which comprises doctoral dissertations, clinical guidelines, and regulatory documents, to uncover any potentially overlooked data.

### 2.2 Inclusion criteria

Inclusion criteria were: (1) participants recruited in China with MDD or another clinically diagnosed depressive disorder in a defined clinical context, including post-stroke, postpartum, perimenopausal, or cancer-related depression, established using the DSM, ICD, CCMD, or another operationalised clinical criterion; symptom descriptions such as ‘depressed mood’ or ‘emotional distress’ alone were insufficient; (2) at least one active arm combined an antidepressant with an acupuncture-related intervention. Needling interventions included manual acupuncture (MA), electroacupuncture (EA), warm needling, fire needling, and auricular acupuncture, whereas moxibustion was treated as a distinct non-needling adjunct; (3) eligible comparators included antidepressant monotherapy, another acupuncture–antidepressant combination, or acupuncture monotherapy; and (4) the study was a randomised controlled trial. In multi-arm studies, sham-acupuncture-plus-antidepressant, psychotherapy-containing, and healthy-control arms were extracted for study description but were not included in the principal efficacy NMA unless they mapped to a prespecified connected node. Blinding and allocation reporting were assessed through RoB 2 rather than used as eligibility requirements. All eligible studies remained in the systematic review, whereas each quantitative NMA was restricted to interventions connected within the corresponding outcome network.

### 2.3 Study selection and data extraction

Independent screening of titles and abstracts was conducted by two reviewers, who also retrieved full texts and assessed eligibility using EndNote. Any disagreements were settled by a third reviewer.

Two independent reviewers (Authors 1 and 2) extracted data using a pre-designed standardized extraction form. The gathered data comprised the following elements: initial author, publication year, nation, research design, characteristics of participants, treatment details (including acupuncture modality and major acupoints), outcome measures, assessment time points, and adverse events (AEs).

When key data were missing or unclear, the original authors were contacted for clarification. Antidepressants were classified by pharmacological mechanism as SSRIs, SNRIs, NaSSAs, or TCAs. Acupuncture-related arms were mapped to MA, EA, sham MA, sham EA, or non-invasive EA as reported. For multi-arm trials, all arms were extracted, but only arms that corresponded to prespecified nodes and formed part of a connected outcome network contributed to the quantitative NMA. Data-extraction discrepancies were resolved by a third reviewer, with consensus reached through group discussion when required.

Inter-rater reliability between the two independent reviewers for full-text screening was high (intraclass correlation coefficient = 0.985; Cohen’s kappa = 0.78), indicating excellent agreement.

### 2.4 Outcomes

The principal efficacy outcome was change in HAMD/HDRS scores. HAMD-17 and HAMD-24 change scores were pooled as raw mean differences in the original score units. Because the two versions have different score ranges, an SMD-based sensitivity analysis was performed to examine whether the choice of scale affected the findings. Other depression instruments, including the SDS, MADRS, BDI, and CGI, were described but were not combined in the principal HAMD network. Safety outcomes were nausea/vomiting, dry mouth, headache, other reported adverse events, and treatment discontinuation for any cause.

### 2.5 Risk of bias

Two reviewers independently assessed the included studies using the Cochrane Risk of Bias 2.0 tool (RoB 2) for randomised trials (6). The domains were the randomisation process, deviations from intended interventions, missing outcome data, outcome measurement, selection of the reported result, and overall risk of bias. Each domain was rated as low risk, some concerns, or high risk. Disagreements were adjudicated by a third reviewer after group discussion.

### 2.6 Statistical analysis

Pairwise meta-analyses and Bayesian NMAs were used to evaluate efficacy and safety. Frequentist pairwise estimates, where reported, used mean differences (MDs) or odds ratios (ORs) with 95% confidence intervals (CIs); Bayesian network estimates used MDs or ORs with 95% credible intervals (CrIs). Change scores were calculated as endpoint minus baseline when they were not reported directly. Missing change-score standard deviations were estimated as SDchange = √(SDbaseline² + SDendpoint² − 2r × SDbaseline × SDendpoint), assuming r = 0.5. Zero-event cells were retained without an additional continuity correction.

For direct comparisons, heterogeneity was examined using Cochran’s Q and I². Values near 25%, 50%, and 75% were interpreted as low, moderate, and high heterogeneity, respectively (7), but model selection was based on clinical and methodological heterogeneity together with the statistical measures rather than on a single I² cut-off. The Bayesian NMA used a random-effects consistency model because variation in clinical context, treatment duration, acupuncture protocol, and antidepressant class was expected across trials (8).

Before NMA, the geometry and connectedness of each outcome network were examined. Nodes represented intervention categories, edge width reflected the number of direct comparisons, and disconnected components were not combined in the same model. Transitivity was considered qualitatively by comparing potential effect modifiers across treatment comparisons, including clinical context, baseline depression severity and scale, treatment duration, antidepressant class, and acupuncture modality. These characteristics were incompletely and unevenly reported; therefore, transitivity could not be established with confidence, and subgroup or meta-regression analyses by clinical context were not undertaken because they would have produced sparse or disconnected networks.

The Bayesian NMA was conducted in R version 3.6.0 using gemtc version 0.8.1. Continuous outcomes used a normal likelihood with identity link, and binary outcomes used a binomial likelihood with logit link. In the absence of a documented non-default specification, the package-default vague priors were reported: relative treatment effects followed normal priors with a standard deviation of 15 times the data-derived outcome-measure scale, and the between-study standard deviation followed a uniform distribution from 0 to that outcome-measure scale. Multi-arm trials were analysed within the gemtc model so that correlations among comparisons from the same trial were retained rather than treated as independent (9).

Four Markov chain Monte Carlo chains were run with 10,000 adaptation/burn-in iterations and 100,000 sampling iterations per chain. Convergence was assessed using the Brooks–Gelman–Rubin diagnostic together with trace and density plots. Model fit was examined using posterior residual deviance and the deviance information criterion. Local inconsistency was assessed by node-splitting for comparisons with both direct and indirect evidence; p < 0.05 was interpreted as evidence of local inconsistency. An SMD-based sensitivity analysis was performed for the HAMD outcome.

Forest plots were used to display pooled network estimates relative to the selected reference treatment. Posterior probabilities of occupying each rank were calculated from the Bayesian model (10). **Table 1** reports rank-specific probabilities from Rank 1 (least effective) to Rank 8 (most effective); these values are not SUCRA scores, and no SUCRA values are reported in this manuscript.

**Table 1:** Probability of treatment ranking for improvement in depressive symptoms.

Intervention	Rank1	Rank2	Rank3	Rank4	Rank5	Rank6	Rank7	Rank8
EA	0.086	0.132	0.205	0.234	0.242	0.082	0.012	0.007
EA_SNRIs	0.191	0.125	0.126	0.170	0.211	0.060	0.075	0.043
EA_SSRIs	0.000	0.000	0.001	0.021	0.121	0.378	0.403	0.076
MA	0.152	0.176	0.205	0.222	0.178	0.056	0.009	0.003
MA_SNRIs	0.000	0.002	0.016	0.043	0.124	0.272	0.138	0.406
MA_SSRIs	0.000	0.000	0.000	0.004	0.030	0.141	0.359	0.466
SNRIs	0.213	0.239	0.225	0.226	0.082	0.011	0.004	0.000
SSRIs	0.359	0.325	0.223	0.080	0.012	0.001	0.000	0.000

Each cell gives the posterior probability that an intervention occupies the specified rank (Rank 1 to Rank 8). Rank 1 is the least effective and Rank 8 the most effective because lower HAMD scores indicate greater improvement. These are rank-position probabilities, not SUCRA values.

Adverse events were summarised using outcome-specific network diagrams and OR-based forest plots. Because several safety networks contained few events and zero-event cells, the posterior ORs and CrIs were interpreted primarily as measures of substantial uncertainty rather than as evidence of a protective effect.

## 3 Results

### 3.1 Study selection

Database searches identified 2,025 records. After removal of 1,014 duplicates, 1,011 records underwent title and abstract screening; 910 were excluded. The full texts of 101 articles were assessed, and 24 were excluded because of another study design (n = 4), ineligible participants (n = 5), unsuitable outcome measures (n = 6), or other concerns (n = 9). Seventy-seven RCTs were included in the systematic review and data synthesis. The selection process is presented in the PRISMA flow diagram (**Figure 1**).

**Figure 1:**
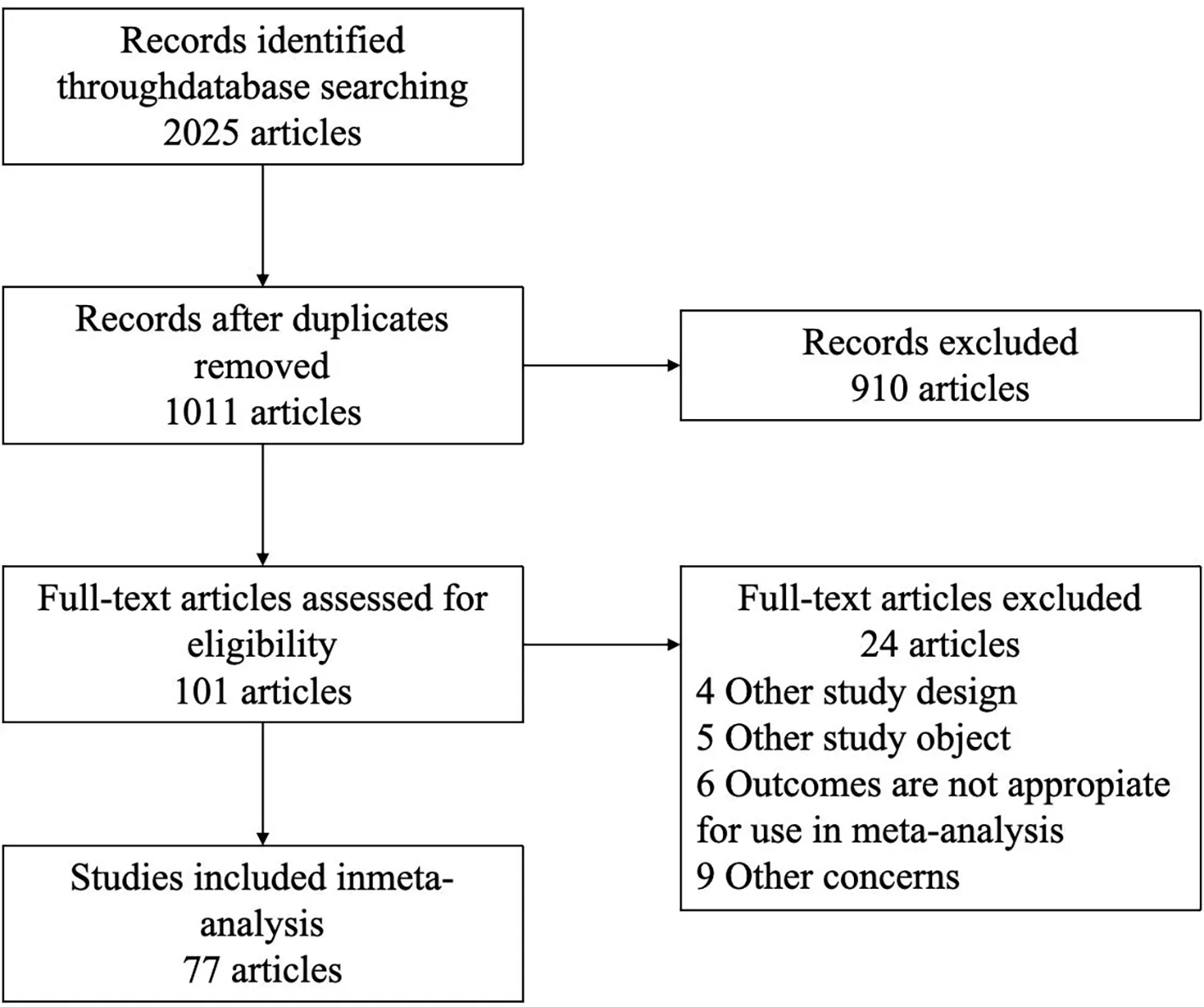
PRISMA flow diagram of study selection.

### 3.2 Characteristics of included studies

Seventy-seven RCTs involving 7,031 participants with MDD or another clinically diagnosed depressive disorder in a defined clinical context were included. All studies were undertaken in China and were published from 2003 to 2025. Sixty-four trials had two arms and 13 had three arms. Diagnostic frameworks included the DSM, ICD, and CCMD; several studies applied more than one framework, so the diagnostic-framework counts are not mutually exclusive. A small number used another or incompletely described criterion. Most trials reported HAMD-17 or HAMD-24, while some used the SDS, BDI, CGI, MADRS, or TESS. Intervention duration ranged from 2 to 12 weeks, most commonly 4 to 8 weeks. Trial populations included primary depression and depression in post-stroke, postpartum, perimenopausal, cancer-related, chronic-pain, and other clinical contexts. The extracted arms included acupuncture–antidepressant combinations, antidepressant monotherapy, acupuncture monotherapy, sham-acupuncture-plus-antidepressant, psychotherapy-containing, and healthy-control arms. Only arms that mapped to the eight connected efficacy nodes contributed to the principal efficacy NMA; the remaining arms were retained for descriptive reporting in **Table 2**.

**Table 2:** Characteristics of the studies included in the systematic review and NMA.

Source	Population			Treatment						Outcome measures
	Diagnosis criteria	No	Age	Intervention group		Comparator group		Third group		
Ai et al, 2018 (11)	DSM-V	I: 50; C: 50	I: 20.1 ± 3.6; C: 20.2 ± 3.5	MA+Paroxetine	MA: 1 time/d, 50 min/time, 6 w; Paroxetine: 20 mg/time, 1 time/d, 6 w	Paroxetine	Paroxetine: 20 mg/time, 1 time/d, 6 w			HAMD-17
Cai et al, 2018 (12)	DSM-IV	I: 39; C: 38	I: 45.27 ± 13.3; C: 46.23 ± 14.5	MA+SSRIs	MA: 2–3 times/w, 6 w; SSRIs: 20–100 mg/d, 6 w	SSRIs	SSRIs: Escitalopram 20 mg/d, Paroxetine 20 mg/d, Sertraline 50 mg/d, Fluvoxamine 100 mg/d, stable dose within 6 w			HAMD-24
Chang et al, 2011 (13)	HAMD-17	I: 50; C: 50	I: 62.4 ± 6.5; C: 63.1 ± 5.8	MA+Fluoxetine	MA: 1 time/d, 6 times/w, 4 w; Fluoxetine: 20 mg/time, 1 time/d, 4 w	Fluoxetine	Fluoxetine: 20 mg/time, 1 time/d, 4 w			HAMD-17
Chen et al, 2011 (14)	CCMD-III	I: 30; C: 30	I: 43.9 ± 11.2; C: 49.0 ± 13.4	MA+Fluoxetine	MA: 1 time/d, 6 times/w, 8 w; Fluoxetine: 20 mg/d, 8 w	Fluoxetine	Fluoxetine: 20 mg/d, 8 w			HAMD-17
Chen et al, 2014 (15)	ICD-10	I: 40; C: 33	I: 40.42 ± 5.65; C: 40.63 ± 5.29	MA+Paroxetine	MA: 3 times/w, 6 w; Paroxetine: 10 mg/time (first 2 d), then 20 mg/time, 1 time/d, 6 w	Paroxetine	Paroxetine: 10 mg/time (first 2 d), then 20 mg/time, 1 time/d, 6 w			HAMD
Dai et al, 2022 (16)	CCMD-III	I: 38; C: 35	I: 48.68 ± 2.28; C: 48.51 ± 3.32	EA+Escitalopram	EA: 1 time/d, 30 min/time, 6 w; Escitalopram: 5–20 mg/d, 6 w	Escitalopram	Escitalopram: start 5 mg/d in the morning, titrate to max 20 mg/d, 6 w			HAMD-17
Duan et al, 2007 (17)	CCMD-III	I: 30; C: 30; T: 30	I: 35.8; C: 33.4; T: 36.3	EA+Fluoxetine	EA: 1 time/d, 6 sessions/course, 1 d rest between courses, 6 courses; Fluoxetine: 20 mg/time, 1 time/d, 6 courses	EA+Psychotherapy	EA: 1 time/d, 6 sessions/course, 1 d rest between courses, 6 courses; Psychotherapy: 2 times/w, 60–90 min/time, 6 w	Fluoxetine+Psychotherapy	Fluoxetine: 20 mg/time, 1 time/d, 6 courses; Psychotherapy: 2 times/w, 60–90 min/time, 6 w	HAMD
Feng et al, 2015 (18)	ICD-10	I: 60; C: 60; T: 60	I: 36.4 ± 9.9; C: 37.1 ± 9.8; T: 36.6 ± 10.0	MA+SSRIs	MA: 1 time/2 d, 6 w; SSRIs: Fluoxetine (20–60 mg/d), Paroxetine (20–60 mg/d), Citalopram (20–60 mg/d), Sertraline (50–200 mg/d), Fluvoxamine (50–300 mg/d), 6 w	SSRIs	SSRIs: Fluoxetine (20–60 mg/d), Paroxetine (20–60 mg/d), Citalopram (20–60 mg/d), Sertraline (50–200 mg/d), Fluvoxamine (50–300 mg/d), 6 w	Healthy control group		MADRS
Gao et al, 2023 (19)	DSM-V	I: 36; C: 35	I: 42 ± 12; C: 38 ± 11	MA+SSRIs	MA: 3 times/w, 1 time/2 d, 6 w; SSRIs: stable dose during study, 6 w	SSRIs	SSRIs: stable dose throughout study, 6 w			HAMD-24
Ge et al, 2007 (20)	CCMD-III	I: 24; C: 24	I: 29.32 ± 8.94; C: 31.92 ± 8.34	EA+Sertraline	EA: 1 time/d, 45 min/time, 5 times/w, 6 w; Sertraline: 50 mg/d, 6 w	Sertraline	Sertraline: 50 mg/d, 6 w			HAMD
Guo et al, 2011 (21)	CCMD-III	I: 32; C: 31	I: 21.5 ± 6.0; T: 21.1 ± 6.0; C: 21.7 ± 5.7	EA+Fluoxetine	EA: 1 time/d, 5 times/w, rest 2 d after 5 d treatment, 6 w; Fluoxetine: 20 mg/time, 1 time/d, 6 w	Fluoxetine	Fluoxetine: 20 mg/time, 1 time/d, 6 w	EA	EA: 1 time/d, 5 times/w, rest 2 d after 5 d treatment, 6 w	HAMD
Guo et al, 2019 (22)	CDDG-2	I: 22; C: 22	I: 40.86 ± 9.84; C: 42.09 ± 10.71	MA+Fluoxetine	MA: 1 time/d, 6 h/time, 3 w; Fluoxetine: 20 mg/d, 3 w	Fluoxetine	Fluoxetine: 20 mg/d, 3 w			HAMD-17
Huang et al, 2010 (23)	CCMD-III	I: 29; C: 29	I: 29.3 ± 8.9; C: 31.9 ± 8.3	MA+SSRIs	MA: 3 times/w, no treatment on Sundays, 6 w; SSRIs: 6 w	SSRIs	SSRIs: individualized initial and adjusted doses based on symptoms and side effects, 6 w			HAMD
Huang et al, 2013 (24)	CCMD-III	I: 30; C: 30	I: 49.25 ± 14.03; C: 50.78 ± 12.96	MA+SSRIs	MA: 3 times/w, 6 w; SSRIs: 20–40 mg/d, 6 w	SSRIs	SSRIs: 20–40 mg/d, 6 w			HAMD
Huang et al, 2014 (25)	CCMD-III	I: 30; C: 30; T: 30	I: 61.43 ± 9.91; C: 62.77 ± 9.32; T: 62.10 ± 8.11	EA+Fluoxetine	EA: 5 times/w, 6 w; Fluoxetine: 20 mg/d, 6 w	Fluoxetine	Fluoxetine: 20 mg/d, 6 w	EA	EA: 5 times/w, 6 w	HAMD-17
Jiang et al, 2008 (26)	CCMD-III	I: 34; C: 34	I: 36.4 ± 11.7; C: 35.6 ± 13.1	MA+Citalopram	MA: 1 time/2 d, 10 times/course, 2 courses; Citalopram: 20 mg/time, 1 time/d, 2 courses	Citalopram	Citalopram: 20 mg/time, 1 time/d, 10 times/course, 2 courses			HAMD
Jiao et al, 2012 (27)	CCMD-III	I: 17; C: 16	I: 63.3; C: 64.2	EA+Paroxetine	EA: 1 time/d, 5 times/w, rest 2 d after 5 d treatment, 28 d; Paroxetine: 20 mg/time, 1 time/d, 28 d	Paroxetine	Paroxetine: 20 mg/time, 1 time/d, 28 d			HAMD-24
Li et al, 2013 (28)	DSM-IV	I: 59; C: 60	I: 33.9 ± 12.0; C: 34.8 ± 12.4	EA+Sertraline	EA: 1 time/d, 6 times/w, 8 w; Sertraline: 50–100 mg/d, 8 w	Sertraline	Sertraline: 50–100 mg/d, 8 w			HAMD-17
Li et al, 2017 (29)	ICD-10	I: 29; C: 28	I: 34 ± 8; C: 35 ± 8	MA+Paroxetine	MA: 1 time/d, 6 times/w, 8 w; Paroxetine: 10–20 mg/d, 8 w	Paroxetine	Paroxetine: 10 mg/d (start), increase to 20 mg/d after 2 d, 8 w			HAMD-17
Li et al, 2017 (30)	ICD-10	I: 99; C: 99	I: 34.6 ± 2.6; C: 34.9 ± 2.3	EA+Paroxetine	EA: 3 times/w, 6 w; Paroxetine: 10–20 mg/d, 6 w	Paroxetine	Paroxetine: 10 mg/time (first 2 d), then 20 mg/time, 1 time/d, 6 w			HAMD-24
Li et al, 2022 (31)	CCMD-3	I: 51; C: 51	I: 56.61 ± 5.49; C: 56.39 ± 5.81	MA+Mirtazapine	MA: 1 time/d, 5 times/w, 6 w; Mirtazapine: 15–30 mg/d, 6 w	Mirtazapine	Mirtazapine: start 15 mg/d, increase to 30 mg/d within 1 w, 6 w			HAMD
Lian et al, 2020 (32)	DSM-5	I: 60; C: 60	I: 62.25 ± 5.43; C: 62.25 ± 5.43	MA+Sertraline	MA: 5 times/w, 6 w; Sertraline: 50 mg/d, 6 w	Sertraline	Sertraline: 50 mg/d, 6 w			HAMD
Liang et al, 2013 (33)	CCMD-III	I: 20; C: 20; T: 20	I: 57 ± 9; C: 53 ± 12; T: 58 ± 8	MA+Blood-letting+Paroxetine	MA: 6 times/w, 4 w; Blood-letting: 3 times/w, 4 w; Paroxetine: 20 mg/time, 1 time/d, 4 w	MA+Paroxetine	MA: 6 times/w, 4 w; Paroxetine: 20 mg/time, 1 time/d, 4 w	Paroxetine	Paroxetine: 20 mg/time, 1 time/d, 4 w	HAMD-17
Lin et al, 2012 (34)	CCMD-III	I: 46; C: 54; T: 44	I: 47 ± 8; C: 47 ± 10; T: 48 ± 10	EA+SSRIs	EA: 3 times/w, 6 w; SSRIs: standard dose, 1 time/d, 6 w	SSRIs	SSRIs: standard oral dose, 1 time/d, 6 w	MA+SSRIs	MA: 3 times/w, 6 w; SSRIs: standard dose, 1 time/d, 6 w	HAMD-17
Lin et al, 2014 (35)	CCMD-III	I: 61; C: 61	I: 41.1 ± 5.7; C: 40.3 ± 6.1	MA+Fluoxetine	MA: 1 time/d, 5 times/w, 4 w; Fluoxetine: 20 mg/d, 4 w	Fluoxetine	Fluoxetine: 20 mg/d, 4 w			HAMD
Liu et al, 2005 (36)	CCMD-III	I: 21; C: 20	I:48.9 ± 12.0: C: 49.0 ± 13.4	EA+SSRIs	EA: 1 time/d, 6 w; SSRIs: standard dose, 6 w	SSRIs	SSRIs: standard dose, 1 time/d, 6 w			HAMD-17
Liu et al, 2014 (37)	CCMD-III	I: 45; C: 45	I: 47.11 ± 8.32; C: 47.58 ± 8.21	MA+SSRIs	MA: 1 time/2 d, 4 w; SSRIs: 10–60 mg/d, 4 w	SSRIs	SSRIs: start 10 mg/d, adjust to 10–60 mg/d after 1 w, 4 w			HAMD-17
Liu et al, 2015 (38)	CCMD-III	I: 45; C: 45	I: 36 ± 11; C: 37 ± 11	MA+SSRIs	MA: 1 time/2 d, 4 w; SSRIs: 20–60 mg/d, 1–2 times/d, adjusted after 1 w, 4 w	SSRIs	SSRIs: start 20 mg/d, adjust to 20–60 mg/d after 1 w, 1–2 times/d, 4 w			HAMD-17
Liu et al, 2016 (39)	CCMD-III	I: 49; C: 48; T: 47	I: 63.08 ± 10.31; C: 64.60 ± 10.35; T: 65.38 ± 9.72	MA+Paroxetine	MA: 6 times/w, 4 w; Paroxetine: 20 mg/d, 4 w	MA+Paroxetine	MA: 6 times/w, 4 w; Paroxetine: 20 mg/d, adjusted after 2–3 w, 4 w	Paroxetine	Paroxetine: 20 mg/d, 4 w	HAMD
Liu et al, 2017 (40)	ICD-10	I: 46; C: 45	I: 47.11 ± 9.10; C: 47.27 ± 9.03	MA+Venlafaxine	MA: 1 time/d, 5 times/w, 8 w; Venlafaxine: 75–225 mg/d, 8 w	Venlafaxine	Venlafaxine: 75–225 mg/d, 8 w			HAMD-17
Liu et al, 2018 (41)	ICD-11	I: 21; C: 21	I: 44 ± 10; C: 43 ± 9	MA+Fluoxetine	MA: 1 time/d (first 3 d), then 1 time/3 d, 8 w; Fluoxetine: 20 mg/time, 1 time/d, 8 w	SA+Fluoxetine	SA: 1 time/d (first 3 d), then 1 time/3 d, 8 w; Fluoxetine: 20 mg/time, 1 time/d, 8 w			MADRS
Liu et al, 2024 (42)	CCMD-III	I: 45; C: 45	I: 49.6 ± 4.0; C: 49.4 ± 3.5	MA+Sertraline	MA: 2 times/w, 1 time/2 d, 6 w; Sertraline: 25 mg/d (first 5 d), then 50 mg/d, 6 w	Sertraline	Sertraline: 25 mg/d (first 5 d), then 50 mg/d, 6 w			HAMD
Ma et al, 2012 (43)	ICD-10	I: 26; C: 26	I: 37.8 ± 10.9; C: 38.2 ± 11.6	MA+Sertraline	MA: 1 time/d, 5 times/w, 6 w; Sertraline: 50 mg/d, 6 w	Sertraline	Sertraline: 50 mg/d, taken after breakfast, 6 w			HAMD-17
Ma et al, 2015 (44)	ICD-10	I: 64; C: 64	I: 39.93 ± 12.93; C: 38.69 ± 14.19	MA+Duloxetine	MA: 5 times/w, 6 w; Duloxetine: 40–60 mg/d, 6 w	Duloxetine	Duloxetine: 40–60 mg/d, 6 w			HAMD
Ma et al., 2011 (45)	ICD-10	I: 25; C: 28	I: 46.27 ± 13.13; C: 40.52 ± 14.21	EA+Paroxetine	EA: 1 time/2 d, 30 min/time, 6 w; Paroxetine: 10 mg/d (first 2 d), then 20 mg/d, 6 w	Paroxetine	Paroxetine: 10 mg/d (first 2 d), then 20 mg/d, 6 w			HAMD-17
Min et al, 2021 (46)	ICD-10	I: 30; C: 30	I: 36.1 ± 9.5; C: 35.4 ± 9.4	MA+Paroxetine	MA: 3 times/w, 6 w; Paroxetine: 10–60 mg/d, 6 w	Paroxetine	Paroxetine: start 10 mg/d, titrate +10 mg/w up to 60 mg/d, 6 w			HAMD
Pan et al, 2014 (47)	CCMD-III	I: 45; C: 45	I: 35.7 ± 11.3; C: 36.2 ± 10.8	MA+Citalopram	MA: 1 time/d, 6 w; Citalopram: 20–40 mg/d, 6 w	Citalopram	Citalopram: 20–40 mg/d, 6 w			HAMD
Qin et al, 2023 (48)	CCMD-III	I: 30; C: 30	I: 36 ± 11; C: 35 ± 12	MA+Sertraline	MA: 1 time/2 d, 6 w; Sertraline: 1 tablet/d, 6 w	Sertraline	Sertraline: 1 tablet/time, 1 time/d, 6 w			HAMD-17
Qu et al, 2013 (49)	ICD-10	I: 54; C: 58; T: 48	I: 32.3 ± 9.6; C: 33.2 ± 9.0; T: 34.4 ± 10.8	MA+Paroxetine	MA: 3 times/w, 6 w; Paroxetine: 10 mg/d (initial), 20 mg/d (from w1), max 40 mg/d, 6 w	EA+Paroxetine	EA: 3 times/w, 6 w; Paroxetine: 10 mg/d (initial), 20 mg/d (from w1), max 40 mg/d, 6 w	Paroxetine	Paroxetine: 10 mg/d (initial), 20 mg/d (from w1), max 40 mg/d, 6 w	HAMD-17
Sun et al, 2012 (50)	CCMD-III	I: 20; C: 20	I: 32.5 ± 10.3; C: 31.5 ± 11.4	EA+Venlafaxine	EA: 1 time/d, 5 times/w, 2 w; Venlafaxine: 75 mg/d (w1), 150 mg/d (w2), 2 w	Venlafaxine	Venlafaxine: 75 mg/d (start), increase to 150 mg/d within 1 w, 2 w			HAMD
Sun et al, 2021 (51)	ICD-10	I: 37; C: 35	I: 49 ± 11; C: 46 ± 12	MA+Sertraline	MA: 6 times/course, 6 courses; Sertraline: 50 mg/d, 6 courses	Sertraline	Sertraline: 50 mg/d, 6 courses			HAMD-24
Tan et al, 2021 (52)	ICD-10	I: 48; C: 49	I: 40.42 ± 5.65; C: 40.63 ± 5.29	MA+Paroxetine	MA: 1 time/2 d, 6 w; Paroxetine: as prescribed, 6 w	Paroxetine	Paroxetine: 1 tablet after breakfast, 1 time/d, 6 w			HAMD-17
Tang et al, 2003 (53)	CCDM-II-R	I: 32; C: 32	I: 31.2; C: 33.6	EA+Amitriptyline	EA: 1 time/d (w1–2), 1 time/2 d (w3–6), 6 w; Amitriptyline: 50 mg/time, 1 time/d, 6 w	Amitriptyline	Amitriptyline: 25 mg/time (day 1), increase by 25 mg/time every other day to 75 mg/time, 1 time/d, duration not specified			SDS
Wang et al, 2010 (54)	ICD-10, CCMD-III	I: 30; C: 29	I: 32.26 ± 8.73; C: 36.22 ± 11.07	EA+Paroxetine	EA: 1 time/2 d, 3 times/w, ≥15 sessions, 30 min/time, 6 w; Paroxetine: 10 mg/d (start), 20 mg/d after 2 d, 6 w	Paroxetine	Paroxetine: 10 mg/d (start), 20 mg/d after 2 d, 6 w			HAMD
Wang et al, 2014 (55)	ICD-9	I: 45; C: 26	I: 22.2 ± 0.60; C: 22.07 ± 0.33	MA+SSRIs	MA: 1 time/d, 5 times/w, 6 w; SSRIs: as prescribed, 6 w	SSRIs	SSRIs: as prescribed, 6 w			HDRS-17
Wang et al, 2016 (56)	ICD-10	I: 18; C: 18	I: 44.5 ± 10.47; C: 43.78 ± 9.10	MA+Fluoxetine	MA: 1 time/d (first 3 d), then 1 time/3 d, 8 w; Fluoxetine: 20 mg/time, 1 time/d, 8 w	SA+Fluoxetine	SA: 1 time/d (first 3 d), then 1 time/3 d, 8 w; Fluoxetine: 20 mg/time, 1 time/d, 8 w			MADRS
Wang et al, 2018 (57)	CCMD-III	I: 39; C: 35	I: 34.2 ± 10.9; C: 33.4 ± 11.8	MA+Fluvoxamine	MA: 5 times on, 2 off, 6 w; Fluvoxamine: 100–150 mg/d, 6 w	Fluvoxamine	Fluvoxamine: 100–150 mg/d, 6 w			HAMD-24
Wang et al, 2019 (58)	DSM-V	I: 30; C: 30	I: 32 ± 8; C: 32 ± 7	MA+Venlafaxine	MA: 3 times/w, 30 min/time, 12 w; Venlafaxine: 75 mg/d (w1), 150 mg/d (w2), 225 mg/d (w3–12), 12 w	Venlafaxine	Venlafaxine: 75 mg/d (w1), 150 mg/d (w2), 225 mg/d (w3–12), 12 w			HAMD-17
Wang et al, 2020 (59)	CCMD-3	I: 50; C: 50	I: 48.4 ± 2.9; C: 49.3 ± 2.9	MA+Fluoxetine	MA: 1 time/d, 20 d; Fluoxetine: 20 mg/d, 20 d	Fluoxetine	Fluoxetine: 20 mg/time, 1 time/d, in the morning, 20 d			HAMD
Wang et al, 2022 (60)	DSM-V	I: 80; C: 80	I: 47.9 ± 6.5; C: 46.3 ± 7.2	MA+Fluoxetine	MA: 5 times/w, 30 min/time, 8 w; Fluoxetine: 20 mg/d, 8 w	Fluoxetine	Fluoxetine: 20 mg/d, 8 w			HAMD
Wu et al, 2010 (61)	CCMD-III	I: 33; C: 33	I: 68.52 ± 4.84; C:69.64 ± 5.19	EA+Citalopram	MA: 5 times/w, 30 min/time, 6 w; Citalopram: 20–40 mg/d, 6 w	Citalopram	Citalopram: 20–40 mg/d, 1 time/d, 6 w			HAMD-17
Wu et al, 2024 (62)	CCMD-III	I: 45; C: 45	I: 66.71 ± 6.80; C: 67.05 ± 7.02	MA+Paroxetine	MA: 3 times/w, 30 min/time, 6 w; Paroxetine: 25 mg/d, 6 w	Paroxetine	Paroxetine: 25 mg/time, 1 time/d, 6 w			HAMD
Xi et al, 2023 (63)	CCMD-III	I: 34; C: 34	I: 37.50 ± 4.15; C: 37.42 ± 4.11	MA+Duloxetine	MA: 5 times/w, rest for 2 d after 5 treatments, 6 w; Duloxetine: 40 mg, 2 times/d, 6 w	Duloxetine	Duloxetine: 40 mg, 2 times/d, 6 w			HAMD-24
Xu et al, 2010 (64)	CCMD-III	I: 25; C: 22; T: 24	I: 44 ± 12; T: 44 ± 13; C: 42 ± 15	EA+Fluoxetine	EA: 1 time/2 d, 30 min/time, 12 w; Fluoxetine: 20 mg/d, 12 w	Fluoxetine	Fluoxetine: 20 mg/d, taken after breakfast, 12 w	EA	EA: 1 time/2 d, 30 min/time, 12 w	HAMD-24
Xu et al, 2011 (65)	CCMD-III	I: 25; C: 25; T: 30	I: 47.51 ± 8.21; C: 48.01 ± 8.14; T: 47.42 ± 8.89	MA+Moxibustion+SSRIs	MA: 3 times/w, 30 min/time, 6 w; Moxibustion: 3 times/w, 30 min/time, 6 w; SSRIs: 20–50 mg/d (based on drug), 6 w	MA+SSRIs	MA: 3 times/w, 1 time/2 d, 30 min/time, 6 w; SSRIs: 20–50 mg/d, 6 w	SSRIs	SSRIs: 20–50 mg/d (based on drug), 6 w	HAMD-17
Xu et al, 2024 (66)	DSM-5	I: 50; C: 50	I: 45.02 ± 10.74; C: 44.76 ± 10.67	MA+Escitalopram	MA: 1 time/2 d, total 15 sessions; Escitalopram: 5–10 mg/d, same duration	Escitalopram	Escitalopram: start 5 mg/d, increase to 10 mg/d within 2–3 w, same duration			HAMD-24
Yan et al, 2023 (67)	DSM-V	I: 30; C: 30	I: 36 ± 11; C: 33 ± 10	MA+Sertraline	MA: 3 times/w, 1 time/2 d, 8 w; Sertraline: 50 mg/d (start), 75–100 mg/d (adjusted at 1–2 w), 8 w	SA+Sertraline	SA: 3 times/w, 1 time/2 d, 8 w; Sertraline: 50 mg/d (start), 75–100 mg/d (adjusted at 1–2 w), 8 w			SDS
Yang et al, 2005 (68)	CCMD-III	I: 34; C: 32	I: 37.5 ± 13.1; C: 34.5 ± 10.3	EA+Fluoxetine	EA: 1 time/d, 6 times/w, 6 w; Fluoxetine: 20 mg/d, 6 w	Fluoxetine	Fluoxetine: start 20 mg/d, increase to 20–40 mg/d within 1 w, 6 w			HAMD
Yang et al, 2015 (69)	CCMD-III	I: 33; C: 30	I: 51.65; C: 53.24	EA+Fluoxetine	EA: 1 time/d, 6 times/w, 6 w; Fluoxetine: 20 mg/time, 1 time/d, 6 w	Fluoxetine	Fluoxetine: 20 mg/time, 1 time/d, 6 w			HAMD-24
Yang et al, 2018 (70)	ICD-10	I: 40; C: 40	I: 34.2 ± 10.9; C: 33.4 ± 11.8	MA+Fluoxetine	MA: 5–6 times/w, 8 w; Fluoxetine: 20 mg/d, 8 w	Fluoxetine	Fluoxetine: 20 mg/d, in the morning, taper over 12 months, 8 w			HAMD
Yang et al, 2019 (71)	ICD-10	I: 40; C: 38	I: 15.15 ± 2.39; C: 15.57 ± 2.86	EA+Sertraline	EA: 12 times/w, 6 w; Sertraline: 100 mg/d, 6 w	Sertraline	Sertraline: 100 mg/d, in the morning, 6 w			HAMD-17
Zhang et al, 2007 (72)	CCMD-III	I: 22; C: 20	I: 36.6 ± 9.7; C: 37.1 ± 10.2	EA+Paroxetine	EA: 1 time/d, 6 times/w, 30 min/time, 6 w; Paroxetine: 10–40 mg/d, 6 w	Paroxetine	Paroxetine: 10–40 mg/d, 6 w			HAMD-17
Zhang et al, 2012 (73)	DSM-IV	I: 36; C: 34	I: 46.3 ± 9.9; C: 48.2 ± 9.8	EA+Fluoxetine	EA: 3 times/w, 9 sessions total; Fluoxetine: start 10 mg/d, titrate to optimal dose within 1 w, max 40 mg/d, 3 w	noninvasiveelectroacupuncture(n-EA)+Fluoxetine	n-EA: 3 times/w, 9 sessions total (30 min/time); Fluoxetine: start 10 mg/d, titrate to max 40 mg/d within 1 w, 3 w			HAMD-17
Zhang et al, 2015 (74)	CCMD-III	I: 30; C: 30	I: 59.4 ± 8.2; C: 61.7 ± 8.4	MA+Fluoxetine	MA: 1 time/d, 4 w; Fluoxetine: 20 mg/d, 4 w	Fluoxetine	Fluoxetine: 20 mg/d, 4 w			HAMD-17
Zhang et al, 2019 (75)	CCMD-III	I: 43; C: 43	I: 58.79 ± 8.53; C: 58.83 ± 8.56	MA+Escitalopram	MA: 5 times/w, 6 w; Escitalopram: 10 mg/d, 6 w	Escitalopram	Escitalopram: 10 mg/time, 1 time/d, 6 w			HAMD
Zhang et al, 2021 (76)	DSM-5	I: 46; C: 46	I: 31 ± 5; C: 32 ± 5	MA+Venlafaxine	MA: 1 time/d, 30 min/time, 6 w; Venlafaxine: 75–150 mg/d, 6 w	Venlafaxine	Venlafaxine: start 75 mg/d, increase to 150 mg/d after 2 w, 6 w			HAMD
Zhang et al, 2021 (77)	CCMD-III	I: 20; C: 20	I: 18.13 ± 6.08; C: 18.57 ± 5.47	MA+Duloxetine	MA: 1 time/d, 30 min/time, 6 times/w; Duloxetine: 1 capsule/d, 6 w	Duloxetine	Duloxetine: 1 capsule/d, 6 w			HAMD
Zhang et al, 2025 (78)	Obstet Gynecol	I: 35; C: 35	I: 30 ± 3; C: 31 ± 4	MA+Venlafaxine	MA: 3 times/w, 4 w/course, 2 courses; Venlafaxine: 75–225 mg/d, 2 courses	Venlafaxine	Venlafaxine: start 75 mg/d, adjust to 75–225 mg/d, 2 courses			HAMD
Zhao et al, 2010 (79)	CCMD-III	I: 48; C: 45	I: 40.9 ± 13.9; C: 41.5 ± 13.9	EA+Paroxetine	EA: 1 time/d, 20 min/time, 3 w; Paroxetine: 10–40 mg/d, 3 w	Paroxetine	Paroxetine: 10–40 mg/d, 3 w			HAMD-17
Zhao et al, 2018 (80)	CCMD-III	I: 55; C: 54; T: 56	I: 64.25 ± 7.53; C: 61.77 ± 7.86; T: 62.84 ± 8.13	MA+Citalopram	MA: 5 times/w, 6 w; Citalopram: 20–60 mg/d, 6 w	Citalopram	Citalopram: start 20 mg/d, increase to 40–60 mg/d as needed, 6 w	MA	MA: 5 times/w, 6 w	HAMD-24
Zhao et al, 2019 (81)	ICD-10	I: 132; C: 125; T: 135	I: 41.42 ± 12.53; C: 41.18 ± 12.00; T: 41.76 ± 12.85	MA+SSRIs	MA: 3 times/w, 30 min/time, 6 w; SSRIs: as prescribed, 6 w	EA+SSRIs	EA: 3 times/w, 30 min/time, 6 w; SSRIs: as prescribed, 6 w	SSRIs	SSRIs: as prescribed, 6 w	HAMD-17
Zhao et al, 2024 (82)	HAMD-17	I: 157; C: 153; T: 155	I: 41.39 ± 12.78; C: 40.75 ± 12.21; T: 41.80 ± 12.78	MA+SSRIs	MA: 3 times/w, 6 w; SSRIs: individualized dose based on severity, 6 w	EA+SSRIs	EA: 3 times/w, 6 w; SSRIs: individualized dose, 6 w	SSRIs	SSRIs: individualized dose based on severity, 6 w	HAMD-17
Zhao et al, 2024 (83)	PSD	I: 25; C: 25	I: 65.08 ± 10.47; C: 66.00 ± 7.31	MA+Sertraline	MA: 6 times/w, rest on Sundays, 30 min/time, 6 w; Sertraline: 50 mg/d, 6 w	Sertraline	Sertraline: 50 mg/d, 6 w			HAMD-17
Zheng et al, 2012 (84)	CCMD-III	I: 44; C: 54	I: 47.11 ± 9.52; C: 48.07 ± 10.09	MA+SSRIs	MA: 1 time/2 d, 3 times/w, 6 w; SSRIs: 20–60 mg/d, 6 w	SSRIs	SSRIs: 20–60 mg/d, 6 w			HAMD
Zhou et al, 2021 (85)	ICD-10, CCMD-III	I: 27; C: 27; T: 28	I: 45 (37, 49); C: 45 (35, 48); T: 47 (40, 50)	MA+Fluoxetine	MA: 1 time/2 d, 6 w; Fluoxetine: 20 mg/d, 6 w	Fluoxetine	Fluoxetine: 20 mg/d, 6 w	MA	MA: 1 time/2 d, 6 w	HAMD
Zhu et al, 2022 (86)	ICD-10	I: 40; C: 40	I: 33 ± 9; C: 32 ± 9	MA+Mirtazapine	MA: 2 times/w, 6 w; Mirtazapine: 30 mg/d, 6 w	Mirtazapine	Mirtazapine: 30 mg/d, 6 w			HAMD
Liu et al, 2013 (36)	CCMD-III	I: 45; C: 45	I: 47 ± 8; C: 48 ± 8	MA+SSRIs	MA: 1 time/2 d, 4 w; SSRIs: as prescribed, 4 w	SSRIs	SSRIs: as prescribed, duration not specified			HAMD-17

I, intervention group; C, comparator group; T, third group; d, day; w, week; min, minutes; h, hours; MA, manual acupuncture; EA, electroacupuncture; SA, sham acupuncture; n-EA, non-invasive electroacupuncture; SSRIs, selective serotonin reuptake inhibitors; HAMD/HRSD/HDRS, the Hamilton Depression Rating Scale (17- or 24-item); MADRS, Montgomery–Åsberg Depression Rating Scale; SDS, Self-Rating Depression Scale; DSM-IV/V/5, Diagnostic and Statistical Manual of Mental Disorders; ICD-9/10/11, International Classification of Diseases; CCMD-III/CCMD-3/CCDM-II-R, Chinese Classification of Mental Disorders (3rd ed./revised 2nd ed.); CDDG-2, Chinese Depression Diagnosis Guideline (2nd ed.); PSD, post-stroke depression; Obstet, Gynecol Obstetrics & Gynaecology (journal).

### 3.3 Acupoint selection

Analysis of the included trials revealed that Baihui (GV20) and Yintang (EX-HN3) constituted the most frequently employed core combination, most often (44 studies) utilized as primary points (12–17, 19–21, 23, 24, 28–30, 33–36, 39, 42, 43, 45, 46, 48–50, 53, 54, 57, 58, 61–65, 68, 72, 73, 78–81, 83, 84). This foundational pair was commonly supplemented with adjunct points, most notably Neiguan (PC6) (25 studies) (12, 13, 18, 19, 23, 24, 29, 33, 39, 42–44, 46, 48–50, 53, 54, 57, 62, 72, 75, 77, 80), Sanyinjiao (SP6)(18 studies) (13, 23, 24, 29, 34, 42–44, 48, 49, 52, 54, 57, 62, 70, 72, 75, 78, 80), Taichong (LR3)(26 studies) (12, 13, 18, 19, 21, 29–33, 39, 40, 42–44, 48, 52, 57, 62, 72, 74–76, 78, 80, 86), and Shenmen (HT7)(16 studies) (13, 18, 32, 33, 39, 40, 42–44, 48, 52, 57, 62, 69, 70, 72, 75, 76, 80). Other frequently reported points included Shenting (GV24)(18 studies) (12–14, 16, 21, 29, 32, 34, 36–38, 51, 55, 74, 76, 84, 87), Fengchi (GB20)(12 studies) (23, 24, 30, 34, 45, 46, 49, 53, 54, 84, 86, 87), and Dazhui (GV14)(16 studies) (14, 23, 24, 30, 31, 33, 34, 37–39, 49, 54, 55, 84, 87, 88), though their application was less uniform across studies. However, the reporting of acupoint selection varied considerably in detail and completeness.

### 3.4 Risk of bias assessment

**Figure 2** summarises risk of bias across the 77 included studies. Sixty-six studies raised concerns regarding the randomisation process, mainly because allocation concealment was inadequately reported. Deviations from intended interventions raised concerns in 59 studies, often because the role and standardisation of acupuncture practitioners were insufficiently described. Fifty-seven studies were rated at low risk for missing outcome data. Outcome measurement raised concerns in 59 studies and was rated high risk in 7 studies. Three studies were at high risk for selection of the reported result.

**Figure 2:**
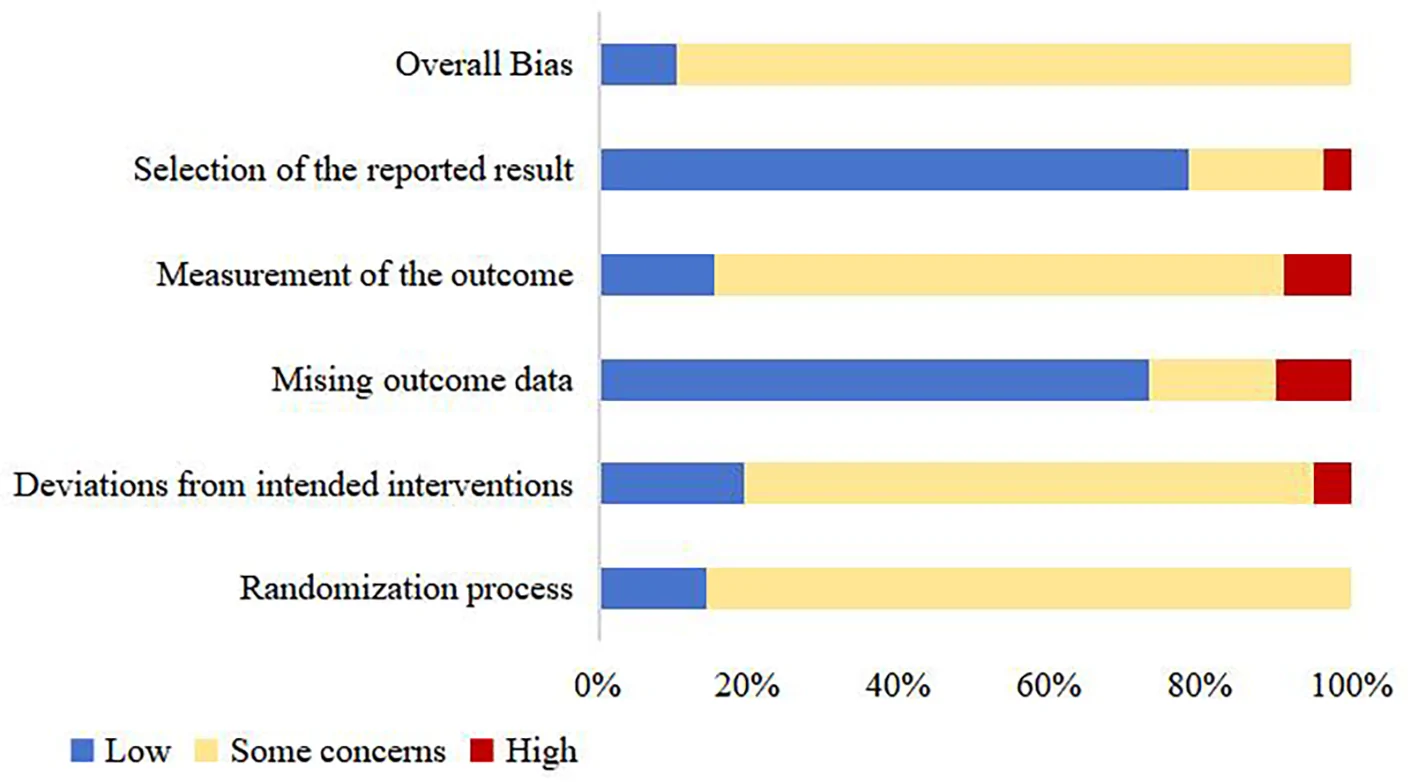
Summary of the risk of bias for the 77 included studies.

### 3.5 Network plot of interventions

**Supplementary Figure 1** presents the efficacy network. The NaSSA and MA + NaSSA evidence formed a disconnected two-node component and was not combined with the principal network. The comparative efficacy NMA therefore comprised eight connected interventions, consistent with **Tables 1**, **3**. The most frequent comparisons were MA + SSRIs versus SSRIs and EA + SSRIs versus SSRIs; MA + SSRIs and SSRIs also contained the largest participant contributions.

**Table 3:** Results of the NMA for all possible treatment comparisons.

Intervention	EA	EA_SNRIs	EA_SSRIs	MA	MA_SNRIs	MA_SSRIs	SNRIs
EA_SNRIs	-0.01 (-4.28, 4.23)	–	–	–	–	–	–
EA_SSRIs	1.93 (-0.05, 3.90)	1.94 (-1.81, 5.72)	–	–	–	–	–
MA	-0.31 (-3.15, 2.52)	-0.29 (-4.59, 4.00)	-2.24 (-4.37, -0.11)	–	–	–	–
MA_SNRIs	2.11 (-1.36, 5.56)	2.13 (-0.98, 5.24)	0.18 (-2.67, 3.02)	2.41 (-1.09, 5.93)	–	–	–
MA_SSRIs	2.32 (0.28, 4.35)	2.34 (-1.45, 6.15)	-0.39 (-1.18, 0.40)	2.63 (0.60, 4.66)	-0.22 (-3.11, 2.67)	–	–
SNRIs	-0.62 (-3.82, 2.56)	-0.61 (-3.41, 2.20)	-2.55 (-5.07, -0.05)	-0.31 (-3.56, 2.94)	-2.73 (-4.06, -1.40)	-2.94 (-5.51, -0.38)	–
SSRIs	-1.05 (-3.04, 0.93)	-1.04 (-4.82, 2.77)	-2.98 (-3.66, -2.31)	-0.74 (-2.78, 1.29)	-3.16 (-6.03, -0.28)	-3.37 (-3.86, -2.89)	0.43 (-2.13, 2.97)

Values are estimated MDs in HAMD scores for each treatment in the column compared with the treatment in the row, with 95% CrIs in parentheses. Negative values favour the treatment in the column. Results are presented in the lower triangle.

### 3.6 Efficacy: NMA

Based on MDs in HAMD scores with 95% CrIs, the NMA (**Table 3**) indicated that MA + SSRIs produced the largest reduction. Relative to SSRIs alone, MA + SSRIs reduced HAMD scores (MD = −3.37, 95% CrI −3.86 to −2.89). EA + SSRIs was also associated with a greater reduction (MD = −2.98, 95% CrI −3.66 to −2.31), as was MA + SNRIs (MD = −3.16, 95% CrI −6.03 to −0.28), although the latter estimate remained relatively imprecise. The CrIs for EA, EA + SNRIs, MA monotherapy, and SNRIs alone included the null.

**Table 3** summarises the NMA estimates for all treatment comparisons. Values are MDs in HAMD scores for each treatment in the column compared with the treatment in the row, with 95% CrIs in parentheses. Negative values favour the treatment in the column. Results are presented in the lower triangle.

#### 3.6.1 Model diagnostics, inconsistency, and sensitivity analysis

The primary efficacy model showed satisfactory convergence. The maximum potential scale reduction factor (PSRF) was 1.00006, and the maximum upper confidence limit was 1.00015. The posterior mean residual deviance (Dbar) was 137.20 for 142 data points (Dbar/data-point ratio = 0.966), with an effective number of parameters (pD) of 100.35 and a deviance information criterion (DIC) of 237.55. These diagnostics were consistent with satisfactory convergence and an acceptable fit to the observed data.

Node-splitting was feasible for five comparisons. There was no evidence of statistically significant local inconsistency for EA + SSRIs versus SNRIs (p = 0.8748), EA + SSRIs versus SSRIs (p = 0.9916), MA + SSRIs versus SNRIs (p = 0.8772), or MA + SSRIs versus SSRIs (p = 0.9696). However, statistically significant local inconsistency was identified for EA + SSRIs versus MA + SSRIs (p = 0.0063). This comparison was therefore interpreted cautiously.

In the SMD sensitivity analysis, EA + SSRIs (SMD = −0.764, 95% CrI −1.030 to −0.498) and MA + SSRIs (SMD = −0.659, 95% CrI −0.842 to −0.476) both favoured combination treatment over SSRIs alone. The direction of effect was consistent with the raw-MD analysis; however, the relative ordering of EA + SSRIs and MA + SSRIs differed. The SMD analysis was therefore treated as a sensitivity check rather than as a basis for treatment ranking.

### 3.7 Network estimates relative to SSRIs

**Figure 3** presents network estimates relative to SSRIs. MA + SSRIs showed an MD of −3.37 (95% CrI −3.86 to −2.89), MA + SNRIs an MD of −3.16 (95% CrI −6.03 to −0.28), and EA + SSRIs an MD of −2.98 (95% CrI −3.66 to −2.31). The CrIs for EA (MD = −1.05, 95% CrI −3.04 to 0.93), EA + SNRIs (MD = −1.04, 95% CrI −4.82 to 2.77), MA (MD = −0.74, 95% CrI −2.78 to 1.29), and SNRIs (MD = −0.43, 95% CrI −2.97 to 2.13) included the null.

**Figure 3:**
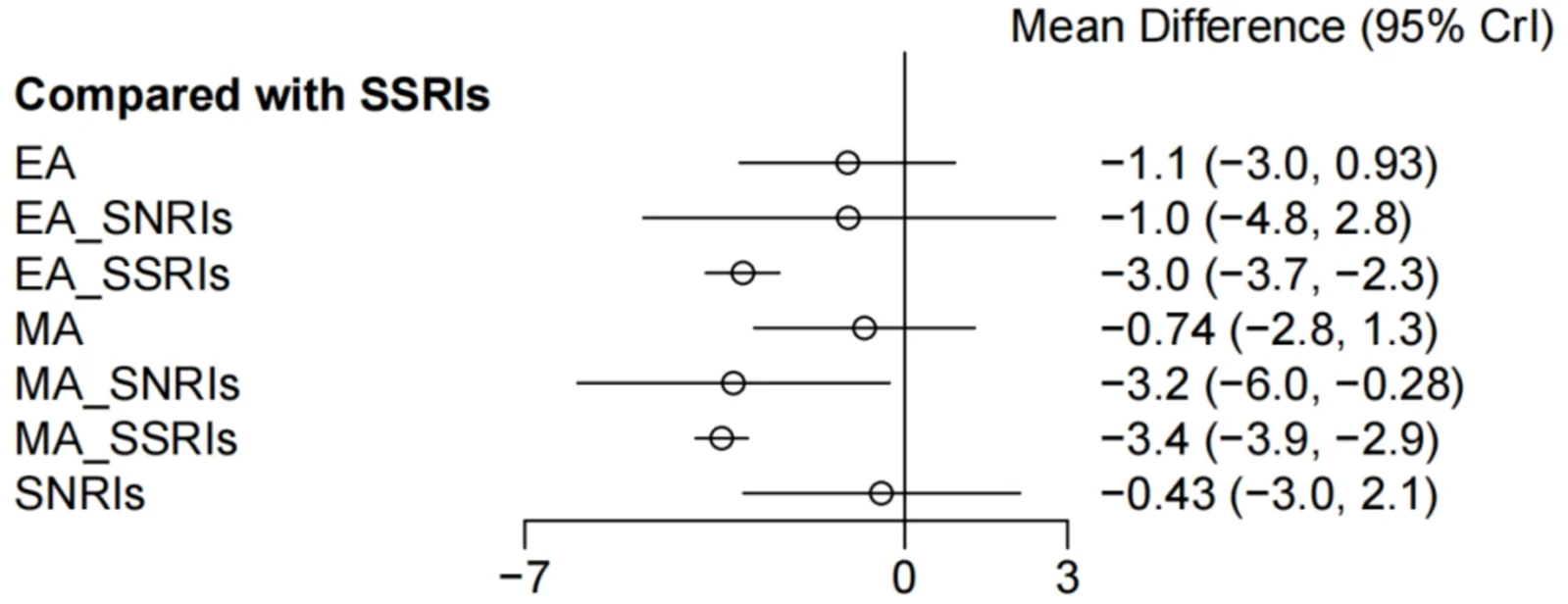
Bayesian network estimates of HAMD mean differences for interventions compared with SSRIs.

### 3.8 Ranking probabilities for efficacy

**Table 1** summarises the posterior probability that each intervention occupies each efficacy rank. Rank 1 denotes the least effective position and Rank 8 the most effective position because lower HAMD scores indicate greater improvement. The values are rank-position probabilities and should not be interpreted as SUCRA scores.

MA + SSRIs had the highest probability of occupying Rank 8 (0.466), followed by MA + SNRIs (0.406) and EA + SSRIs (0.076). The corresponding Rank 8 probability for SSRIs monotherapy was 0.000. These probabilities describe uncertainty in rank position and do not establish clinical superiority on their own.

### 3.9 Adverse effects: NMA

Adverse events included nausea/vomiting, dry mouth, and headache. Treatment discontinuation for any cause could not be quantitatively analysed because few studies reported this outcome and the available data were insufficient for a meaningful synthesis.

#### 3.9.1 Nausea and vomiting

**Supplementary Figure 2** presents the nausea/vomiting network, and **Figure 4** presents ORs for each intervention relative to SSRIs. Extremely small OR estimates were observed for SNRIs (OR = 2.1 × 10^-5^), EA + SNRIs (OR = 6.5 × 10^-6^), and MA + SNRIs (OR = 5.6 × 10^-6^). The corresponding 95% CrIs spanned many orders of magnitude, indicating severe imprecision. These values are therefore interpreted as sparse-data estimates influenced by zero-event cells rather than as evidence of very large protective effects.

**Figure 4:**
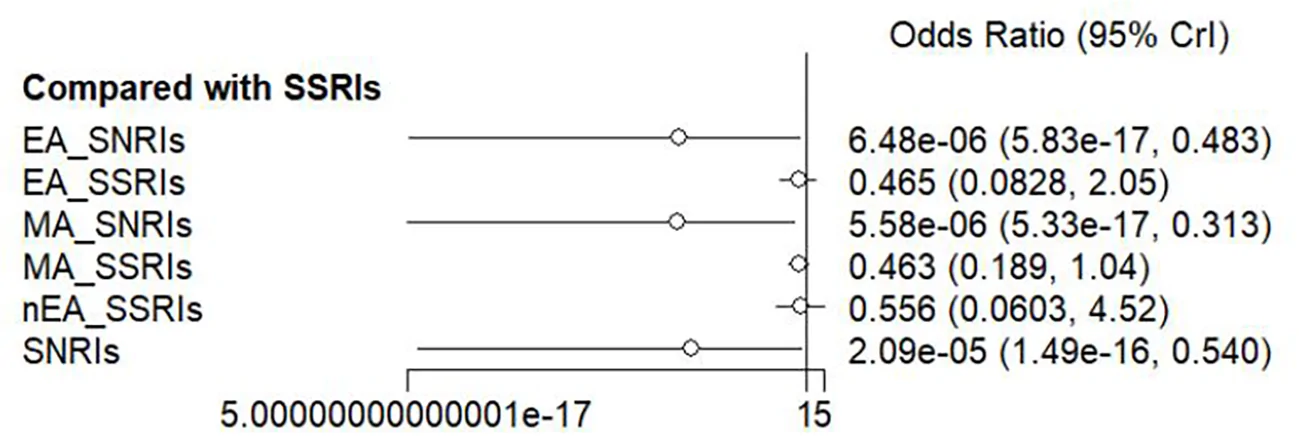
Bayesian network estimates of nausea/vomiting odds ratios for interventions compared with SSRIs.

#### 3.9.2 Dry mouth and headache

**Supplementary Figure 3** presents the dry-mouth network and **Figure 5** the corresponding ORs relative to SSRIs. **Supplementary Figure 4** presents the headache network and **Figure 6** the corresponding ORs. For both outcomes, the 95% CrIs for the acupuncture–antidepressant interventions included 1.0. Given the small number of events and wide CrIs, the findings indicate an absence of a statistically clear difference rather than evidence of equivalent safety.

**Figure 5:**
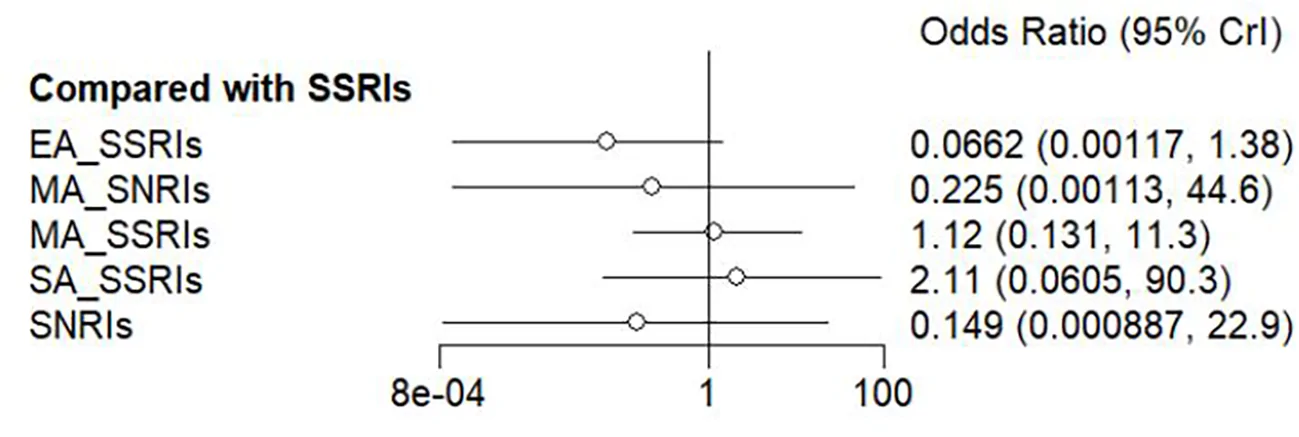
Bayesian network estimates of dry-mouth odds ratios for interventions compared with SSRIs.

**Figure 6:**
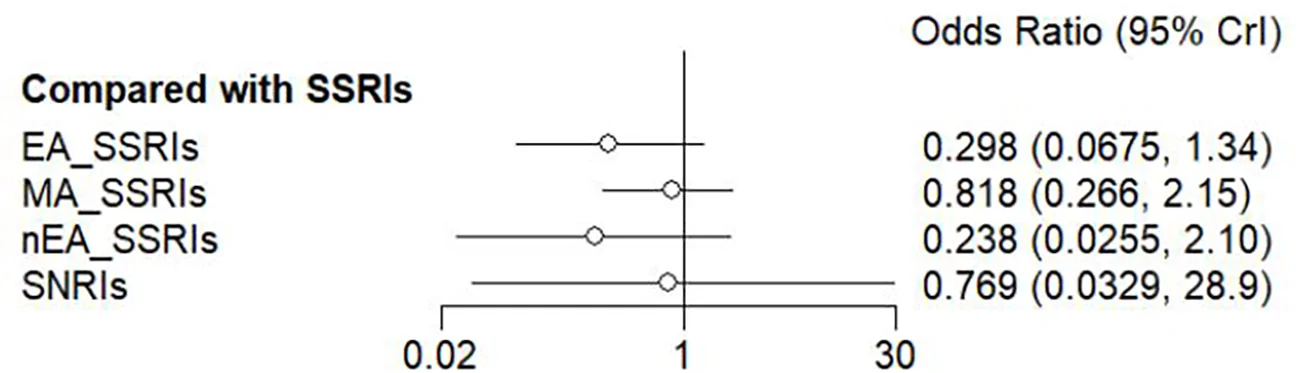
Bayesian network estimates of headache odds ratios for interventions compared with SSRIs.

## 4 Discussion

### 4.1 Treatment efficacy

Across 77 RCTs conducted in China, MA + SSRIs produced the largest short-term HAMD reduction within the principal connected network (MD = −3.37, 95% CrI −3.86 to −2.89). It also had the highest probability of occupying Rank 8 (0.466), followed by MA + SNRIs (0.406) and EA + SSRIs (0.076). These values are probabilities of occupying the most effective rank, not SUCRA scores. The SMD sensitivity analysis retained the direction of benefit for both EA + SSRIs and MA + SSRIs relative to SSRIs, although their relative ordering differed from the raw-MD analysis. Ranking probabilities should be interpreted together with effect magnitude, CrI width, risk of bias, and the plausibility of the transitivity assumption.

The direction of the findings is broadly consistent with earlier meta-analyses that reported greater symptom improvement when acupuncture was added to antidepressant treatment (89). Chen et al. (90) identified EA + SSRI as the most efficacious option, whereas MA + SSRI had the highest Rank 8 probability in the present network. Differences in included populations, publication periods, node definitions, and network structure may explain the different ranking patterns; the available evidence does not support a definitive comparison between the two reviews.

Given the use of acupuncture in Chinese clinical practice, the estimates provide preliminary information on adjunctive treatment options. They do not establish MA + SSRI as a preferred first-line strategy, particularly because the trials were clinically heterogeneous, generally short term, and frequently raised risk-of-bias concerns.

### 4.2 Biological plausibility and proposed mechanisms

Preclinical and neuroimaging studies suggest that acupuncture may influence serotonergic neurotransmission, HPA-axis regulation, neuroimmune signalling, and prefrontal–limbic connectivity (91–94). These pathways are biologically compatible with, but do not prove, an interaction with antidepressant pharmacotherapy. Any mechanistic explanation should therefore be regarded as supportive context rather than evidence that the clinical combination is synergistic.

Other proposed pathways include modulation of tryptophan metabolism and the gut–brain–immune axis, inhibition of TLR4/NF-κB, NLRP3, and cGAS–STING signalling, and promotion of BDNF-related neuroplasticity and neurotransmitter balance (103–113). Most evidence for these pathways is preclinical, and it cannot establish the mechanism or magnitude of the treatment effects observed in this NMA.

### 4.3 Acupoint selection and its rationale

The acupoint regimens identified in the included trials were grounded in traditional Chinese medicine theory and were also discussed in relation to modern neuroanatomical hypotheses (95–97). Baihui (GV20) and Yintang (EX-HN3) formed the most common core combination. In TCM, GV20 is used to raise clear Yang and calm the spirit, while Yintang is used to tranquilise the mind and relieve mental distress (93, 97–100). From a neuroanatomical perspective, stimulation at these sites has been hypothesised to influence prefrontal–limbic circuits involved in mood regulation, although the clinical relevance of this mechanism remains uncertain.

The core pair was frequently supplemented with Neiguan (PC6), Shenmen (HT7), Sanyinjiao (SP6), and Taichong (LR3), reflecting TCM pattern differentiation aimed at calming the spirit, regulating Liver qi, and nourishing Blood (101, 102). This individualised approach may increase clinical relevance, but variation in point selection and manipulation also contributes to between-trial heterogeneity and limits reproducibility.

### 4.4 Safety profile

The available safety networks did not show statistically clear increases in nausea/vomiting, dry mouth, or headache for MA + SSRIs or EA + SNRIs relative to SSRIs; however, many CrIs were extremely wide. The very small ORs for some interventions are likely to reflect sparse events and zero-event cells rather than precise protective effects. Treatment discontinuation was also reported too infrequently for quantitative synthesis. Accordingly, the findings should not be interpreted as demonstrating equivalent safety. Future trials should use standardised adverse-event definitions, report denominators and zero events explicitly, and provide complete discontinuation data.

### 4.5 Strengths and limitations

This review incorporated a large Chinese evidence base, compared eight interventions within the principal connected efficacy network, and examined both efficacy and safety. Nevertheless, the findings are constrained by study quality. Allocation concealment and practitioner-related procedures were often inadequately reported, outcome measurement raised frequent concerns, and acupuncture protocols varied in point selection, manipulation, treatment frequency, and reporting completeness. These limitations reduce reproducibility and may contribute to between-study heterogeneity.

Clinical heterogeneity is also important. The evidence included primary depression as well as post-stroke, postpartum, perimenopausal, cancer-related, chronic-pain, and other depressive disorders. Potential effect modifiers were unevenly distributed and incompletely reported, so transitivity remains uncertain. HAMD-17 and HAMD-24 were pooled using raw MDs to retain the original scale units, but their different ranges may affect comparability; the SMD sensitivity analysis retained the direction of benefit for both EA + SSRIs and MA + SSRIs relative to SSRIs, although their relative ordering differed from the raw-MD analysis. Node-splitting identified local inconsistency for EA + SSRIs versus MA + SSRIs (p = 0.0063), warranting caution for this comparison. Comparison-specific GRADE ratings were not available for this revision. Safety reporting was incomplete and event counts were sparse. Most trials lasted less than eight weeks and all were undertaken in China, limiting inference about long-term outcomes and other healthcare settings. These limitations require cautious interpretation of both effect estimates and rank probabilities.

### 4.6 Clinical implications

For patients with a clinically diagnosed depressive disorder who have an inadequate response to SSRI monotherapy, MA may be considered as a possible adjunct after individual assessment of diagnosis, treatment preference, contraindications, access, and practitioner competence. The present results do not justify designating a particular acupuncture–antidepressant combination as first-line treatment or directly incorporating a ranking into clinical guidelines. Recent Chinese integrated-medicine guidance permits consideration of acupuncture as an adjunct (114), but comparative effectiveness, acceptability, and cost-effectiveness require better-quality evidence.

### 4.7 Future research

Future research should prioritise multicentre randomised trials with adequate allocation concealment, blinded outcome assessment, follow-up of at least six months, and complete adverse-event and discontinuation reporting. Trials should distinguish primary MDD from depression in specific clinical contexts and report baseline severity, HAMD version, antidepressant dose, acupuncture dose, and STRICTA intervention details. Prespecified analyses should assess transitivity, local and global inconsistency, alternative outcome standardisation, prior sensitivity, and comparison-specific certainty of evidence. Studies outside East Asia and formal cost-effectiveness comparisons with antidepressant dose escalation or switching are also needed.

## 5 Conclusion

Within the connected short-term efficacy network, MA + SSRIs was associated with the largest HAMD reduction and the highest probability of occupying the most effective rank. This finding does not establish a preferred first-line strategy or equivalent safety. Clinical heterogeneity, risk-of-bias concerns, uncertain transitivity, sparse adverse-event data, and limited follow-up mean that the result should be considered provisional until it is confirmed in rigorous, longer-term trials.

## Data Availability

The original contributions presented in the study are included in the article/**Supplementary Material**. Further inquiries can be directed to the corresponding authors.

## Supplementary material

Supplementary Figure 1 Network plot of evidence (efficacy).

Supplementary Figure 2 Network plot of evidence for nausea/vomiting.

Supplementary Figure 3 Network plot of evidence for dry mouth.

Supplementary Figure 4 Network plot of evidence for headache.

Supplementary Appendix 1 Search strategy (Cochrane library).

Supplementary Table 1 Retrieval strategy in Cochrane.

Supplementary Appendix 2 Search strategy (Embase).

Supplementary Table 2 Retrieval strategy in Embase.

Supplementary Appendix 3 Search strategy (PubMed).

Supplementary Table 3 Retrieval strategy in PubMed.

Supplementary Appendix 4 Search strategy (Web of Science).

Supplementary Table 4 Retrieval strategy in Web of Science.

Supplementary Appendix 5 Search strategy (CNKI).

Supplementary Table 5 Retrieval strategy in CNKI.

Supplementary Appendix 6 Search strategy (Wanfang).

Supplementary Table 6 Retrieval strategy in Wanfang.
