# Supplementary material for: Acupuncture–antidepressant combinations for clinically diagnosed depressive disorders in China: a systematic review and network meta-analysis
Source: Front Psychiatry. 2026 Oct 20;17:1805745. doi: 10.3389/fpsyt.2026.1805745 (PMC13727528; doi:10.3389/fpsyt.2026.1805745)
Supplement: Supplementary file 1 [file DataSheet1.pdf]

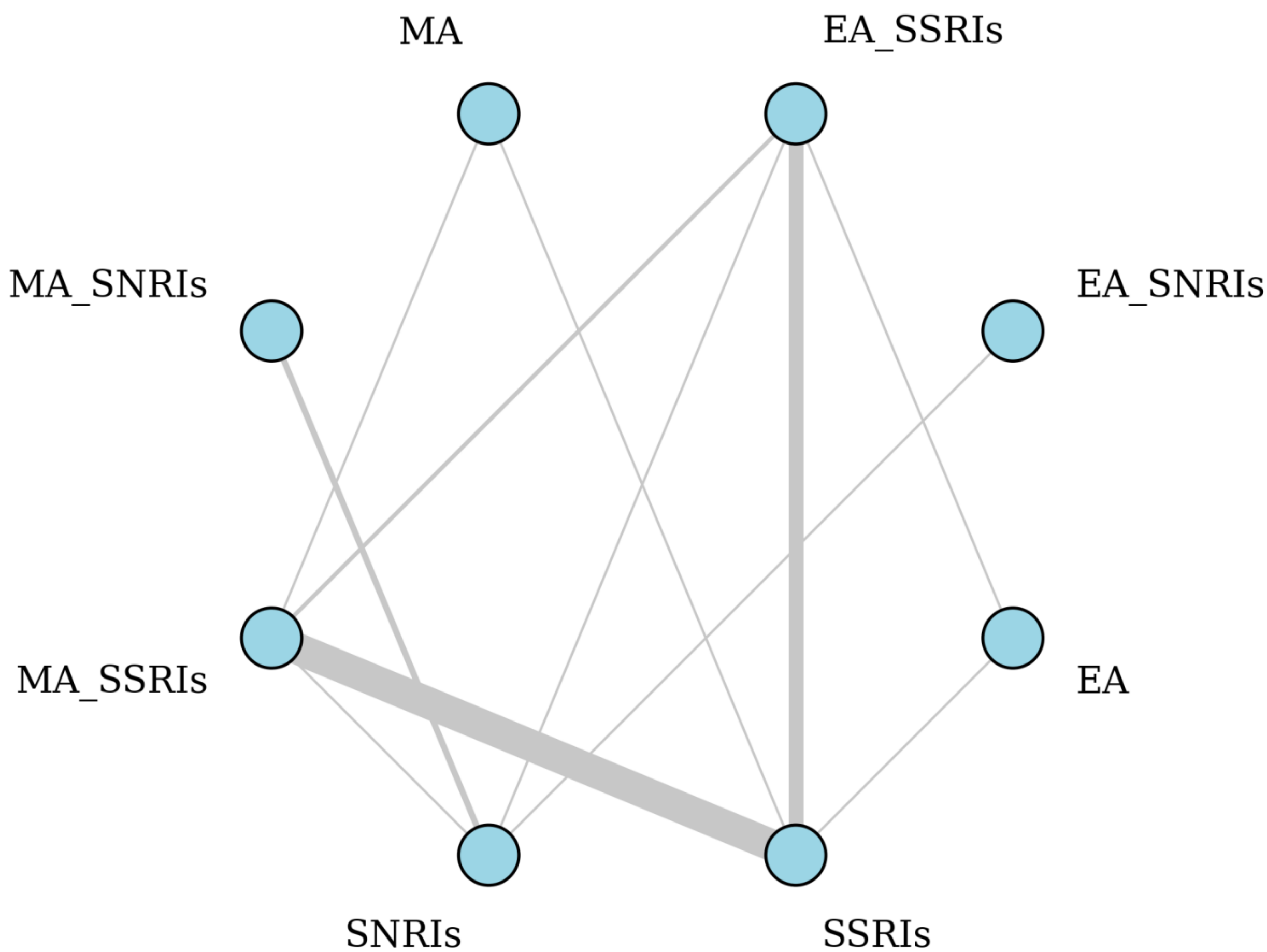

Supplementary Fig. 1 Network plot of evidence (efficacy)

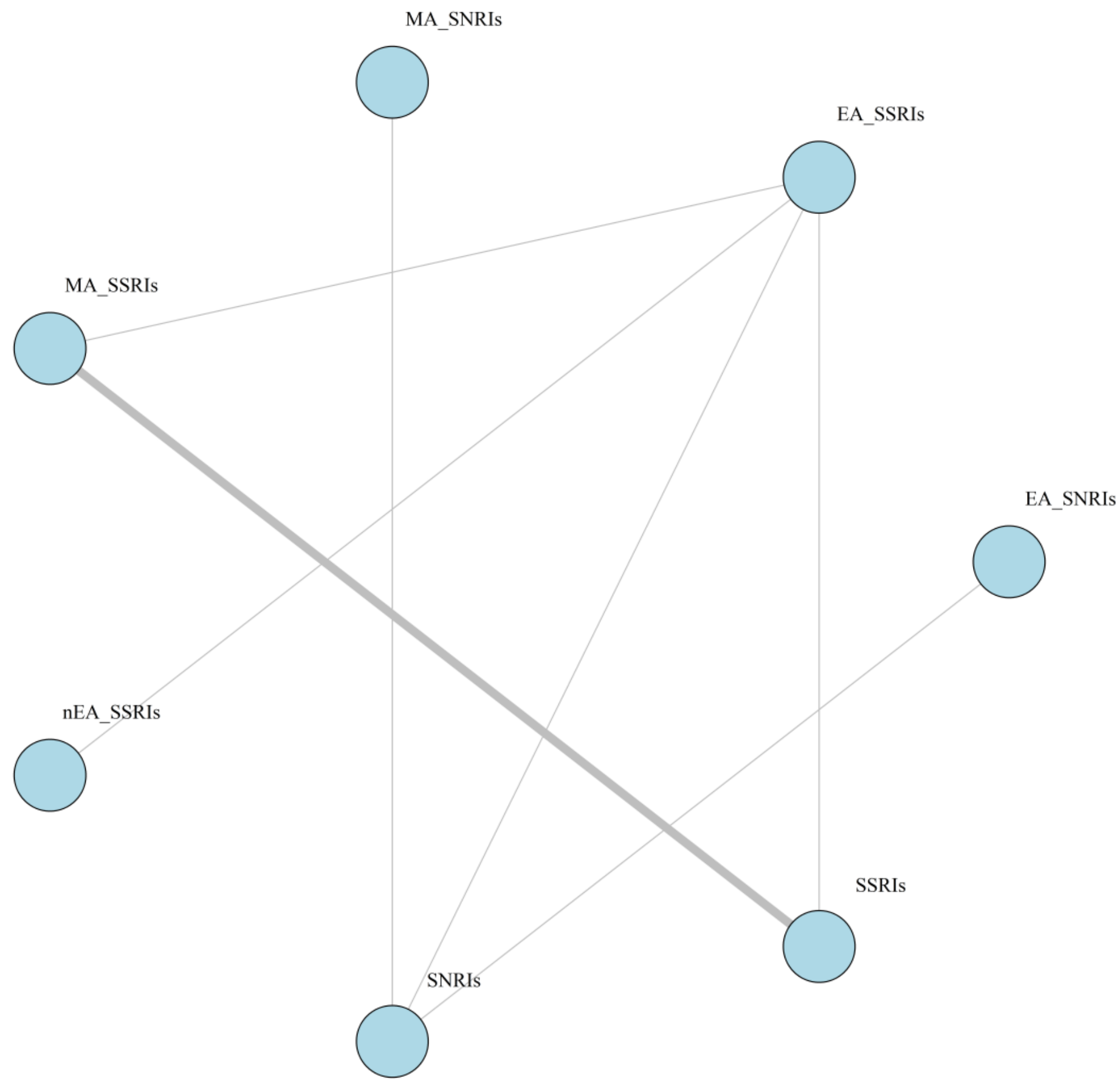

Supplementary Fig. 2 Network plot of evidence for nausea/vomiting

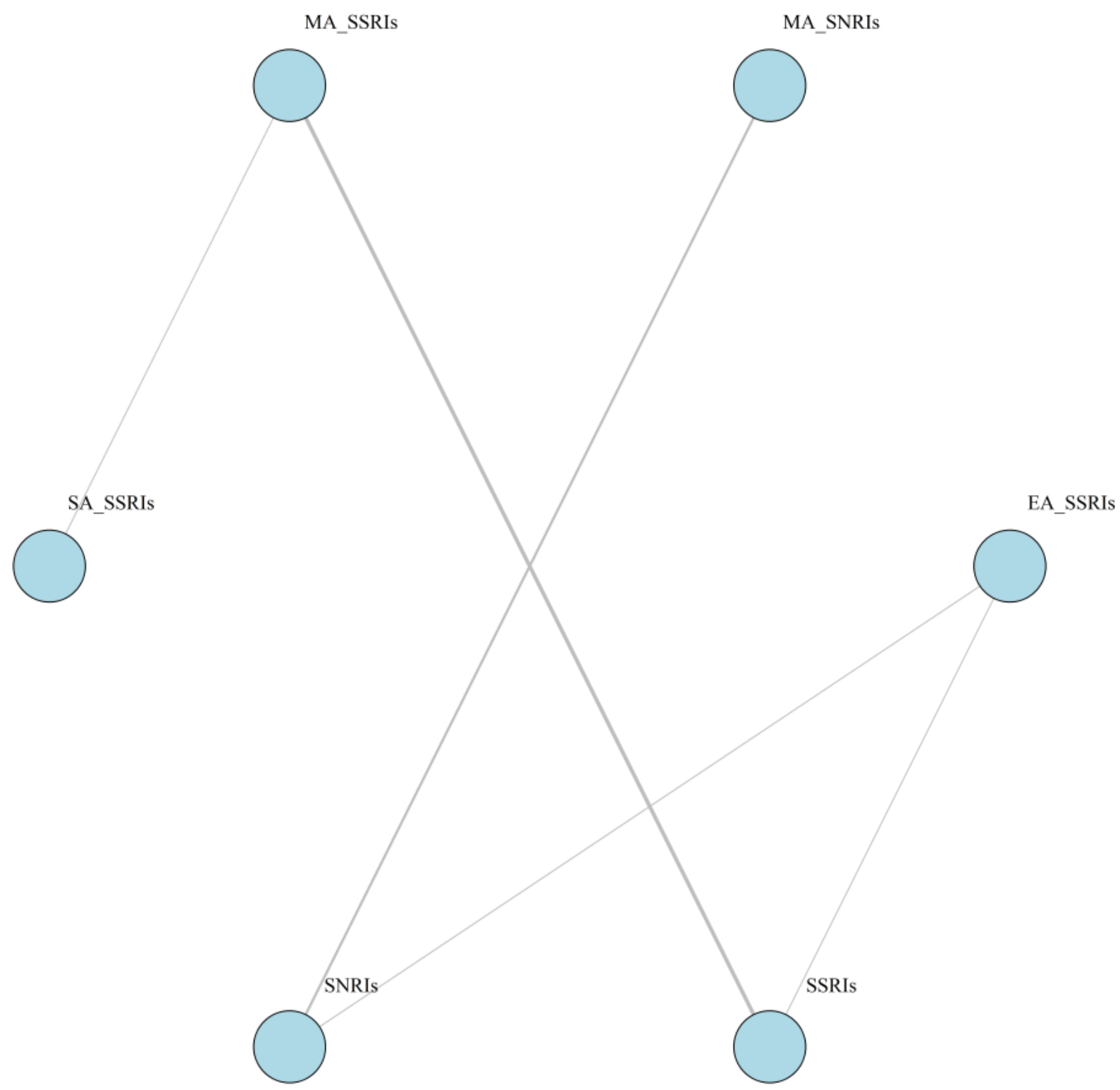

Supplementary Fig. 3 Network plot of evidence for dry mouth

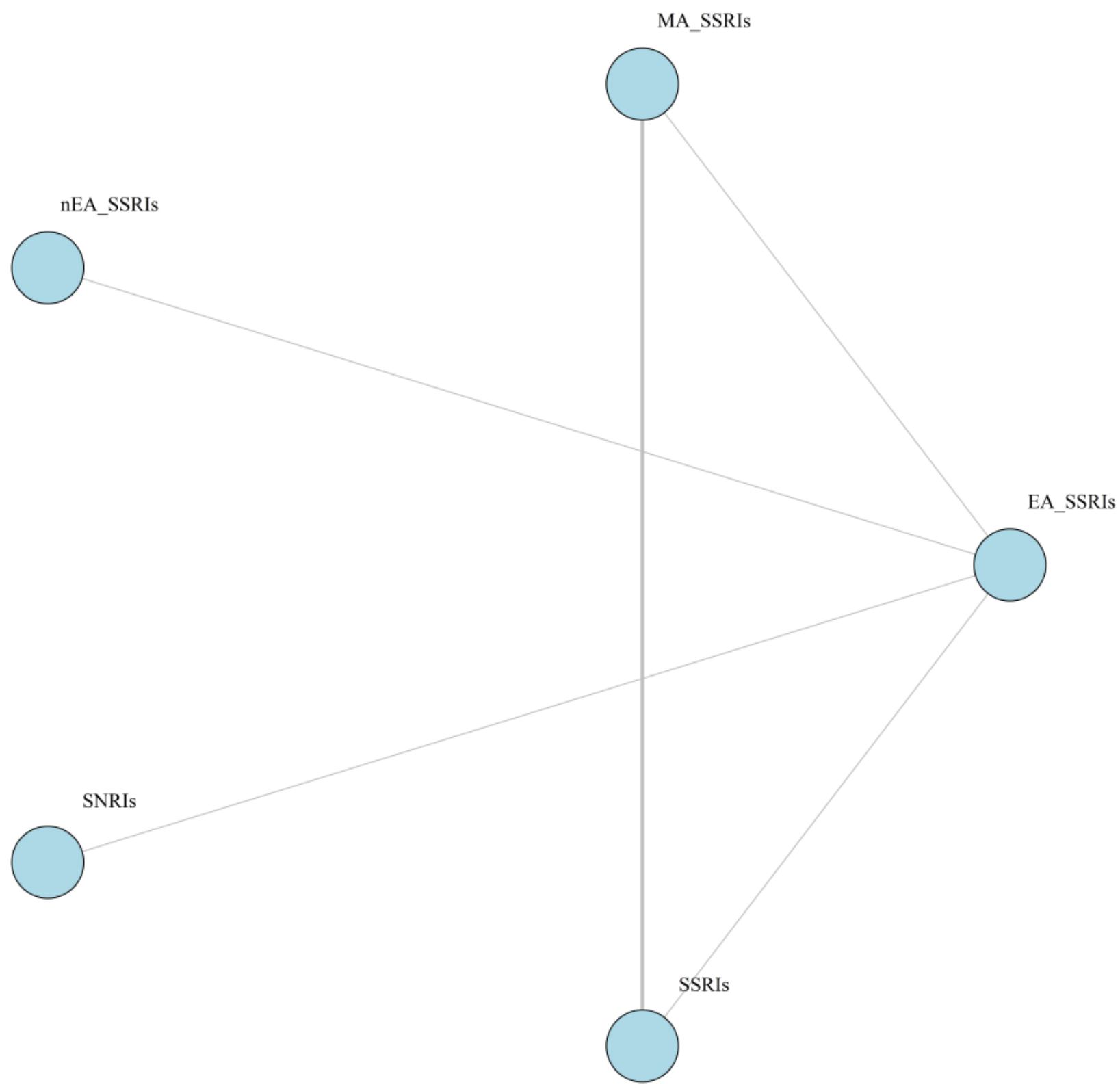

Supplementary Fig. 4 Network plot of evidence for headache

Search Strategy

Supplementary Appendix 1. Search strategy (Cochrane Library)

Table S1. Retrieval strategy in Cochrane

#	Searches
1	depressi*
2	acupuncture or electroacupuncture or warm needling or fire needling or auricular acupuncture or auricular points or moxibustion or acupoints
3	SSRIs or Fluoxetine or Paroxetine or Sertraline or Fluvoxamine or Citalopram or Escitalopram
4	SNRIs or Venlafaxine or Duloxetine or Levomilnacipran
5	NaSSA or Mirtazapine
6	TCAs or Amitriptyline or Clomipramine
7	#3 or #4 or #5 or #6
8	#1 and #2 and #7

Supplementary Appendix 2. Search strategy (Embase)

Table S2. Retrieval strategy in Embase

#	Searches
1	('acupuncture':ti,ab,kw OR 'electroacupuncture':ti,ab,kw OR 'warm needling':ti,ab,kw OR 'fire needling':ti,ab,kw OR 'auricular acupuncture':ti,ab,kw OR 'auricular points':ti,ab,kw OR 'moxibustion':ti,ab,kw OR 'acupoints':ti,ab,kw)
2	depressi*
3	('SSRIs':ti,ab,kw OR 'Fluoxetine':ti,ab,kw OR 'Paroxetine':ti,ab,kw OR 'Sertraline':ti,ab,kw OR 'Fluvoxamine':ti,ab,kw OR 'Citalopram':ti,ab,kw OR 'Escitalopram':ti,ab,kw OR 'SNRIs':ti,ab,kw OR 'Venlafaxine':ti,ab,kw OR 'Duloxetine':ti,ab,kw OR 'Levomilnacipran':ti,ab,kw OR 'NaSSA':ti,ab,kw OR

	'Mirtazapine':ti,ab,kw OR 'TCAs':ti,ab,kw OR 'Amitriptyline':ti,ab,kw OR 'Clomipramine':ti,ab,kw)
4	trial:ti,ab,kw
5	#1 and #2 and #3 and #4

Supplementary Appendix 3. Search strategy (PubMed)

Table S3. Retrieval strategy in PubMed

#	Searches
1	"Depressive Disorder"[MeSH] OR depressi*[Title/Abstract]
2	"Acupuncture Therapy"[MeSH] OR "Moxibustion"[MeSH] OR acupuncture[Title/Abstract] OR electroacupuncture[Title/Abstract] OR "warm needling"[Title/Abstract] OR "fire needling"[Title/Abstract] OR "auricular acupuncture"[Title/Abstract] OR "auricular points"[Title/Abstract] OR moxibustion[Title/Abstract] OR acupoints[Title/Abstract]
3	"Serotonin Uptake Inhibitors"[MeSH] OR Fluoxetine[Title/Abstract] OR Paroxetine[Title/Abstract] OR Sertraline[Title/Abstract] OR Fluvoxamine[Title/Abstract] OR Citalopram[Title/Abstract] OR Escitalopram[Title/Abstract]
4	"Serotonin and Noradrenaline Reuptake Inhibitors"[MeSH] OR Venlafaxine[Title/Abstract] OR Duloxetine[Title/Abstract] OR Levomilnacipran[Title/Abstract]
5	Mirtazapine[MeSH Terms] OR Mirtazapine[Title/Abstract]
6	"Tricyclic Antidepressive Agents"[MeSH] OR Amitriptyline[Title/Abstract] OR Clomipramine[Title/Abstract]
7	#3 OR #4 OR #5 OR #6
8	#1 AND #2 AND #7

Supplementary Appendix 4. Search strategy (Web of Science)

Table S4. Retrieval strategy in Web of Science

#	Searches
1	(TS=(depress* OR "depressive disorder" OR "major depressive disorder" OR "treatment-resistant depression"))
2	TS=("acupuncture" OR "electroacupuncture" OR "warm needling" OR "fire needling" OR "auricular acupuncture" OR "auricular points" OR "moxibustion" OR "acupoints")
3	TS=("SSRIs" OR "Fluoxetine" OR "Paroxetine" OR "Sertraline" OR "Fluvoxamine" OR "Citalopram" OR "Escitalopram" OR "SNRIs" OR "Venlafaxine" OR "Duloxetine" OR "Levomilnacipran" OR "NaSSA" OR "Mirtazapine" OR "TCAs" OR "Amitriptyline" OR "Clomipramine" OR "antidepressant*")
4	TS=(random* or controll*)
5	#1 AND #2 AND #3 AND #4

Supplementary Appendix 5. Search strategy (CNKI)

Table S5. Retrieval strategy in CNKI

#	Searches
1	TKA=(针刺 OR 针灸 OR 电针 OR 温针 OR 火针 OR 耳针 OR 耳穴 OR 艾灸 OR 穴位) AND TKA=(5羟色胺 OR SSRIs OR 氟西汀 OR 帕罗西汀 OR 舍曲林 OR 氟伏沙明 OR 西酞普兰 OR 艾司西酞普兰 OR 5羟色胺和去甲肾上腺素再摄取抑制剂 OR SNRIs OR 文拉法辛 OR 度洛西汀 OR 左旋米那普仑 OR 米氮平 OR 三环类OR 阿米替林 OR 氯米帕明) AND TKA=(临床 AND 随机)

Supplementary Appendix 6. Search strategy (Wanfang)

Table S6. Retrieval strategy in Wanfang

#	Searches
1	(摘要:针刺 OR 摘要:针灸 OR 摘要:电针 OR 摘要:温针 OR 摘要:火针 OR 摘要:耳针 OR 摘要:耳穴 OR 摘要:艾灸 OR 摘要:穴位) AND (摘要:5羟色胺 OR 摘要:SSRIs OR 摘要:氟西汀 OR 摘要:帕罗西汀 OR 摘要:舍曲林 OR 摘要:氟伏沙明 OR 摘要:西酞普兰 OR 摘要:艾司西酞普兰 OR 摘要:"5羟色胺和去甲肾上腺素再摄取抑制剂" OR 摘要:SNRIs OR 摘要:文拉法辛 OR 摘要:度洛西汀 OR 摘要:左旋米那普仑 OR 摘要:米氮平 OR 摘要:三环类 OR 摘要:阿米替林 OR 摘要:氯米帕明) AND 摘要:临床 AND 随机
